# The prognostic impact of preoperative blood monocyte count in pathological T3N0M0 rectal cancer without neoadjuvant chemoradiotherapy

**DOI:** 10.1007/s13277-015-3560-6

**Published:** 2015-05-21

**Authors:** Lu-Ning Zhang, Weiwei Xiao, Pu-Yun OuYang, Kaiyun You, Zhi-Fan Zeng, Pei-Rong Ding, Zhi-Zhong Pan, Rui-Hua Xu, Yuan-Hong Gao

**Affiliations:** 1 Department of Radiation Oncology, Sun Yat-sen University Cancer Center, State Key Laboratory of Oncology in South China, Collaborative Innovation Center for Cancer Medicine, No. 651 Dongfeng Road East, Guangzhou, 510060 Guangdong China; 2 Department of Colorectal Surgery, Collaborative Innovation Center for Cancer Medicine, Guangzhou, Guangdong China; 3 Department of Medical Oncology, Sun Yat-sen University Cancer Center, State Key Laboratory of Oncology in South China, Collaborative Innovation Center for Cancer Medicine, Guangzhou, Guangdong China; 4 Department of Oncology, The Second Affiliated Hospital of Sun Yat-sen University, Guangzhou, China

**Keywords:** Monocyte count, Prognosis, Pathological T3N0M0, Rectal cancer

## Abstract

It remains controversial whether adjuvant therapy should be delivered to pathological T3N0M0 rectal cancer without neoadjuvant chemoradiotherapy. Thus identification of patients at high risk is of particular importance. Herein, we aimed to evaluate whether the absolute peripheral blood monocyte count can stratify the pathological T3N0M0M0 rectal cancer patients in survival. A total of 270 pathological T3N0M0 rectal cancer patients with total mesorectal excision-principle radical resection were included. The optimal cut-off value of preoperative monocyte count was determined by receiver operating characteristic curve analysis. Overall survival and disease-free survival between low- and high-monocyte were estimated by Kaplan–Meier method and Cox regression model. The optimal cut-off value for monocyte count was 595 mm^3^. In univariate analysis, patients with monocyte counts higher than 595/mm^3^ had significantly inferior 5-year overall survival (79.2 vs 94.2 %, *P* = 0.006) and disease-free survival (67.8 vs 86.0 %, *P* < 0.001). With adjustment for the known covariates, monocyte count remained to be associated with poor overall survival (HR = 2.55, 95 % CI 1.27–5.10; *P* = 0.008) and disease-free survival (HR = 2.63, 95 % CI 1.48–4.69; *P* = 0.001). Additionally, the significant association of monocyte count with disease-free survival was hardly influenced in the subgroup analysis, whereas this correlation was restricted to the males and patients with normal carcinoembryonic antigen (CEA) level (<5 μg/L), tumor grade II, and with adjuvant therapy. High preoperative monocyte count is independently predictive of worse survival of pathological T3N0M0 rectal cancer patients without neoadjuvant chemoradiotherapy. Postoperative adjuvant therapy might be considered for patients with high-monocyte count.

## Introduction

Rectal cancer accounts for approximately 28 % of all colorectal malignancies [1, 2]. Albeit that neoadjuvant chemoradiotherapy (CRT) followed by total mesorectal excision (TME) surgery is now recommended for clinical stage II/III rectal cancer, it remains controversial whether this is applicable to patients with clinical stage T3N0. Due to the great CRT expenses and increased toxicities and other causes, it is a common phenomenon in China that parts of patients with clinical stage T3N0 rectal cancer fail to receive neoadjuvant CRT at the initial time. Additionally, some patients with clinical stage T1–2N0 may be upstaged with pathological T3N0M0 after radical resection. Thus, it is the question whether postoperative adjuvant treatment should be delivered to those patients who avoid preoperative chemoradiotherapy and are staged with pathological T3N0 after complete radical resection. Considering the inaccuracy of imaging, only pathological diagnosed T3N0 after resection were entered into this study. As postoperative adjuvant chemotherapy is recommended to rectal cancer staged with T4N0M0 (stage II) according to ESMO guideline, these sort of patients are excluded for analysis. Since the current evidence of significant advantage of neoadjuvant over adjuvant chemoradiotherapy in the whole stage II/III group of patients [3], it is not ethical to conduct a randomized controlled trial to investigate whether adjuvant treatment could be safely omitted in this special group of patients at the present time. Therefore, in the absence of definite knowledge of the benefit from adjuvant treatment for T3N0 disease in the TME era, it is of particular importance to identify high-risk patients to obtain survival gains from adjuvant treatment and low-risk patients to free from adjuvant treatment toxicities and expenses.

Evidence indicates that cancer-associated inflammation plays a key role in the development and survival of a broad range of cancers [4]. Peripheral blood monocyte is the key immune cell in the inflammatory response, and has been independently associated with the prognosis of various malignancies, such as diffuse large B cell lymphoma [5], hepatocellular carcinoma [6], cervical cancer [7], metastatic melanoma [8], and lung adenocarcinoma [9]. However, it is still unknown if monocyte can predict the survival of patients with rectal cancer. This study assessed the prognostic impact of preoperative monocyte count on the survival of pathological T3N0M0 patients.

## Material and methods

### Patients

This retrospective study was approved by the Institutional Review Board at Sun Yat-sen University Cancer Center, and individual informed consent was waived given the anonymous analysis of routine data. A total of 270 rectal cancer patients who underwent TME between June 2004 and November 2011 were included. All patients were pathologically staged T3N0M0 after surgery. Moreover, all patients received complete preoperative evaluation, including endorectal ultrasound, computed tomography (CT), magnetic resonance imaging (MRI), chest radiography, blood count, and liver function test. Baseline monocytes count was established from peripheral blood samples within 1 week before the initiation of any treatment modality. To exclude the influence of various comorbidities or other disease states, all included patients had no self-reported acute infections or rectal disorders, indicating that the cell counts could represent the baseline value.

### Treatment

Surgical resection was defined as radical when there was no evidence of distant metastases and tumor clearance was both macroscopically and histologically complete. All the operations were carried out according to the TME-principles by colorectal surgeons, and the methods included low anterior resection (LAR) and abdominoperineal resection (AR).

### Follow-up

Patients were examined every 3 months during the first 2 years, with follow-up examinations every 6 months thereafter. Evaluations included complete blood count, liver function test, CEA, CA19-9, physical examination, and digital rectal examination. Chest radiography, CT scanning of the abdomen and pelvis, and colonoscopy were conducted every 6 months after surgery.

### Statistical analysis

The optimal cut-off value of monocyte count was determined using receiver operating characteristic (ROC) curve analysis. At each value, sensitivity and specificity were plotted, thus generating an ROC curve. The score closest to the point with both maximum sensitivity and specificity was selected as the cut-off value. Covariates balance between low- and high-monocyte groups were examined by *t* test (continuous variable), *χ* ^2^ test, or Fisher’s exact test (categorical variable) as appropriate.

The main endpoints were overall survival (OS) and disease-free survival (DFS), defined as the time of surgery to the date of death from all causes, and to the date of either locoregional recurrence, distant metastasis, respectively. OS and DFS rates were estimated with Kaplan–Meier method and log-rank test. Multivariate analysis was performed by Cox proportional hazards regression with backward LR method. Two-sided *P* < 0.05 was considered to be statistically significant. All statistical analyses were performed by SPSS software, version20.

## Results

### Patients

The median follow-up of all patients was 53 months (range, 3–106 months). There were 19 (7.0 %) cases of locoregional relapse, 37 (13.7 %) cases of distant metastasis, and 32 (11.9 %) cases of death, respectively. The 3- and 5-year OS was 94.1 and 89.8 %, respectively, and the 3- and 5-year DFS was 90.1 and 84.5 %, respectively.

**Fig. 1:**
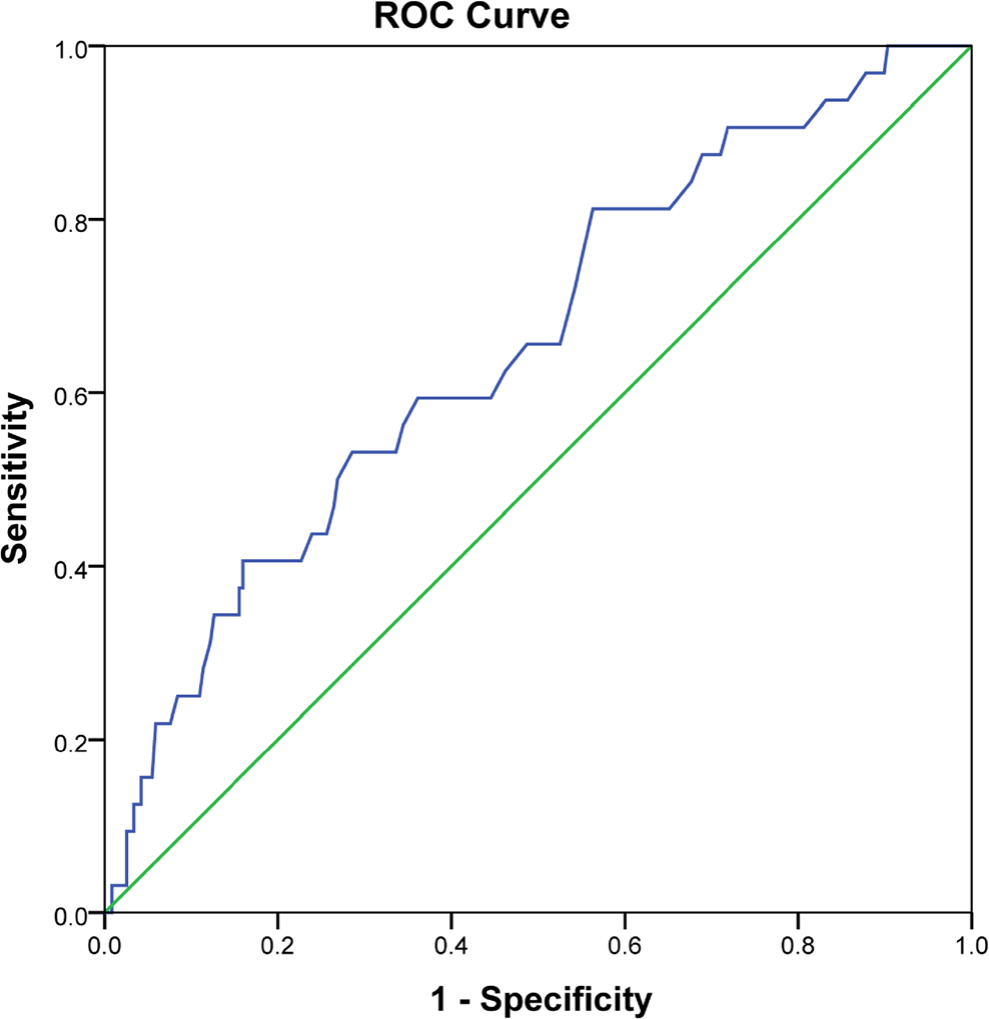
Receiver operating characteristic analysis

According to the ROC curve analysis (Fig. 1), patients were stratified into low- and high-monocyte group by the optimal cut-off value of 595/mm^3^. The clinicopathological characteristics were summarized in Table 1. Obviously, high-monocyte count (≥595/mm^3^) was significantly associated with male (*P* < 0.001), elevated CEA level (≥5 μg/L) (*P* = 0.016), and high tumor grade (*P* = 0.031). However, patients with low-monocyte count (<595/mm^3^) were quite similar to those with high-monocyte count in age, tumor location, operation, and adjuvant chemotherapy.

**Table 1:** Comparison of clinicopathological characteristics between all the pT3N0M0 patients with low- and high-monocyte counts

Characteristics	<595/mm^3^		≥595/mm^3^		*P*
	No.	%	No.	%	
Sex					<0.001
Male	102	55.1	66	77.6	
Female	83	44.9	19	22.4	
Age (years)					0.808
<61	90	48.6	40	47.1	
≥61	95	51.4	45	52.9	
CEA level					0.016
Normal	118	63.8	41	48.2	
Elevated	67	36.2	44	51.8	
Tumor location					0.772
≤5 cm	51	27.6	22	25.9	
>5 cm	134	72.4	63	74.1	
Operation					0.158
AR	151	81.6	63	74.1	
APR	34	18.4	22	25.9	
Tumor grade					0.031
I	9	4.9	10	11.8	
II	164	88.6	65	76.5	
III	12	6.5	10	11.8	
Adjuvant therapy					0.361
No	58	31.4	22	25.9	
Yes	127	68.6	63	74.1	

*AR* anterior resection, *APR* abdominoperineal resection

### OS and DFS according to monocyte count

**Fig. 2:**
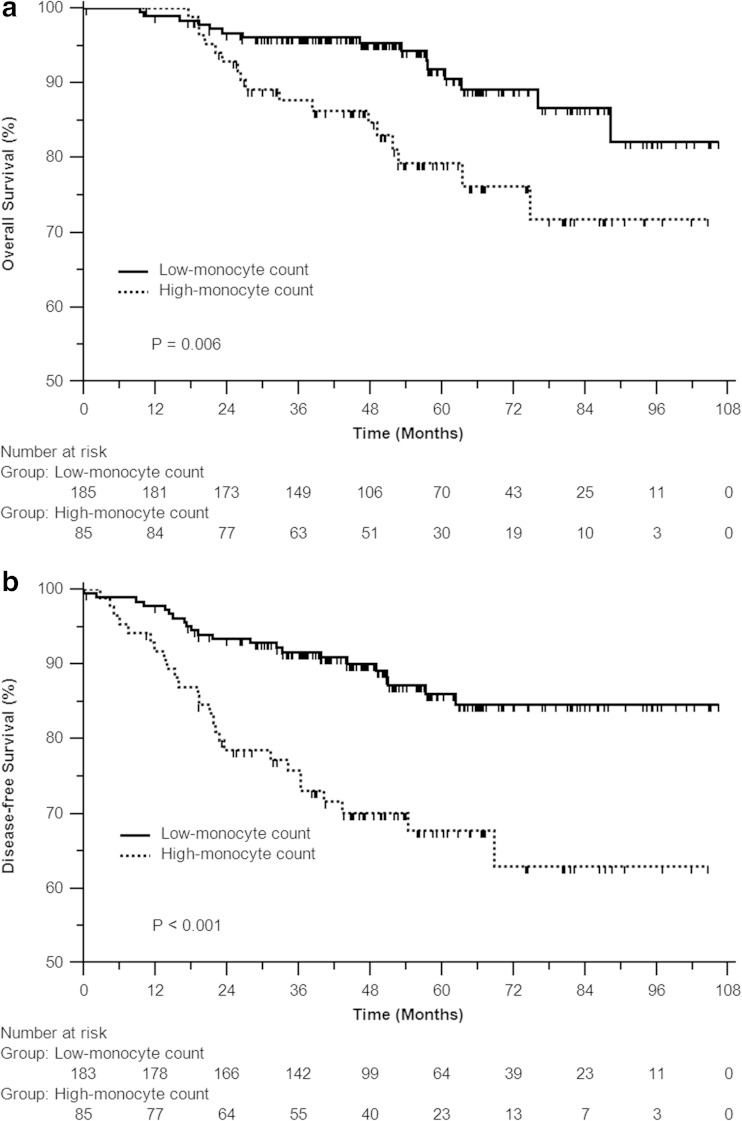
Overall survival (**a**) and disease-free survival (**b**) of patients with low- and high-monocyte counts

The 3- and 5-year OS rate was 96.1 and 94.2 % for patients with low-monocyte count, and 87.7 and 79.2 % for those with high-monocyte count. Correspondingly, the 3- and 5-year DFS rate was 91.6 and 86 % for patients with low-monocyte count, and 75.8 and 67.8 % for those with high-monocyte count, respectively. Apparently, patients with low-monocyte count had significantly superior OS and DFS over those with high-monocyte count (*P* = 0.006 and *P* < 0.001, respectively, Fig. 2a, b).

Adjusting for the known covariates including sex, age, CEA level, tumor location, operation, tumor grade, and adjuvant chemotherapy, patients with high-monocyte count still showed significantly higher risk of death (HR = 2.55, 95 % CI 1.27–5.10; *P* = 0.008) and disease (HR = 2.63, 95 % CI 1.48–4.69; *P* = 0.001) than those with low-monocyte count (Table 2).

**Table 2:** Univariate and multivariate analysis of potential prognostic factors

	Overall survival			Disease-free survival		
	Unadjusted P	Adjusted HR (95 % CI)	Adjusted *P*	Unadjusted *P*	Adjusted HR (95 % CI)	Adjusted *P*
Monocyte (<595/mm^3^ vs ≥595/mm^3^)	0.006	2.55 (1.27–5.10)	0.008	<0.001	2.63 (1.48–4.69)	0.001
Sex (male vs female)	0.115	0.63 (0.28–1.41)	0.258	0.046	0.59 (0.30–1.14)	0.117
Age (<61 vs ≥61 years)	0.530	1.23 (0.60–2.52)	0.581	0.069	1.65 (0.91–3.00)	0.096
CEA level (normal vs elevated)	0.704	1.01 (0.49–2.09)	0.977	0.011	1.72 (0.96–3.08)	0.070
Tumor location (≤5 vs >5 cm)	0.999	0.81 (0.30–2.14)	0.664	0.291	0.67 (0.37–1.24)	0.202
Operation (AR vs APR)	0.959	0.89 (0.38–2.08)	0.796	0.885	0.52 (0.22–1.21)	0.128
Tumor grade (I/II/III)	0.936	0.84 (0.39–1.81)	0.662	0.977	0.98 (0.50–1.93)	0.945
Adjuvant therapy (no vs yes)	0.574	0.76 (0.36–1.62)	0.482	0.885	0.88 (0.46–1.71)	0.713

*AR* anterior resection, *APR* abdominoperineal resection, *HR* hazard ratio, *CI* confidence interval

### Subgroup analysis

Given the above positive association of monocyte count with sex, CEA level, and tumor grade, there might be possible interactions between them. To clarify the influence, thus, we did the subgroup analysis (Table 3). In the male patients, monocyte count was independently associated with both OS (HR = 2.85, 95 % CI 1.24–6.51; *P* = 0.013) and DFS (HR = 2.73, 95 % CI 1.39–5.37; *P* = 0.004). However, the prognostic impact of monocyte count was observed for DFS (HR = 4.69, 95 % CI 1.44–15.34; *P* = 0.011), but not for OS (HR = 1.55, 95 % CI 0.29–8.24; *P* = 0.606) among the females. Similarly, the associations of monocyte count with DFS were observed in all the patients regardless of the CEA level; nevertheless, the significant relations of monocyte count with OS were only obtained in the strata of patients with normal CEA level. Additionally, monocyte count was significantly correlated with OS and DFS among patients with tumor grade II, instead of grades I and III. And for patients with adjuvant therapy, monocyte count was the prognostic factor for OS and DFS, whereas for those with operation alone, monocyte count was just independently predictive of DFS.

**Table 3:** Subgroup analysis of the association between monocyte count and overall survival and disease-free survival

Subgroup	Overall survival					Disease-free survival				
	No. at risk/No. of events		HR	95 % CI	P*	No. at risk/No. of events		HR	95 % CI	P*
	Low- MO	High-MO				Low-MO	High-MO			
Sex										
Male	102/9	66/15	2.85	1.24–6.51	0.013	102/15	66/21	2.73	1.39–5.37	0.004
Female	83/6	19/2	1.55	0.29–8.24	0.606	83/7	19/5	4.69	1.44–15.34	0.011
CEA level										
Normal	118/8	41/9	3.38	1.31–8.78	0.012	118/10	41/10	3.96	1.60–9.78	0.003
Elevated	67/7	44/8	1.49	0.54–4.13	0.441	67/12	44/16	2.26	1.07–4.80	0.033
Tumor grade										
I	9/2	10/1	–		0.960	9/3	10/1	1.40	0.05–40.79	0.844
II	164/12	65/14	3.05	1.41–6.59	0.005	164/18	65/22	2.91	1.54–5.50	0.001
III	12/1	10/2	–		0.874	12/1	10/3	4.63	0.24–88.40	0.309
Adjuvant therapy										
No	58/6	22/4	1.35	0.35–5.14	0.662	58/7	22/7	3.20	1.05–9.75	0.041
Yes	127/9	63/13	3.26	1.39–7.64	0.007	127/15	63/19	2.97	1.51–5.86	0.002

*HR* hazard ratio, *CI* confidence interval, *MO* monocyte count

*Adjusted for sex, age (<61 vs ≥61 years), CEA level (<5 vs ≥5 μg/L), tumor location (≤5 vs >5 cm), operation, tumor grade, and adjuvant chemotherapy except the factor defined as the stratum

## Discussion

To our knowledge, this is the first large-scale study to evaluate the prognostic significance of the absolute monocyte count in pathological T3N0M0 rectal cancer patients treated with TME resection and without undergoing preoperative chemoradiotherapy. We demonstrated that high-monocyte count independently predicted worse survival of patients. This finding was highly consistent with those in hepatocellular carcinoma [6], cervical cancer [7], metastatic melanoma [8], and lung adenocarcinoma [9].

Unfortunately, the biological reasons behind this correlation remain speculative but previously published experimental studies support the observation. Firstly, it may relate to the insidious progression of cancer disease involving activation of innate immunity [10]. Monocytes play a key role in innate immunity, constitute nearly 5 % of the circulating white blood cell pool, and exhibit a short half-life in the circulation of a few hours [11]. The presence of immunocompetent cells within tumors is assumed to reflect the immunological antitumor response. Secondly, the causal link between inflammation and cancer is now well established [12, 13]. Monocytes, known as the key component of inflammation system, might directly stimulate cancer cell growth by producing various proinflammatory cytokines, such as interleukin-1, interleukin-6, and tumor necrosis factor. In addition, monocytes can be actively attracted to the tumor site and differentiate into tumor-associated macrophages (TAMs) via the cytokines and chemokines produced by tumor cells, such as monocyte chemoattractant protein-1 and vascular endothelial growth factor [14, 15]. Thus, the circulating level of monocytes may reflect formation or presence of TAMs. Many macrophage-released soluble factors directly stimulate the growth of tumor cells and promote tumor cell migration and metastasis [12, 16, 17]. Macrophages can produce enzymes and inhibitors that regulate the digestion of the extracellular matrix, hence favoring tumor invasion and migration [18, 19] and can also contribute to tumor progression by secretion of factors that enhance neoangiogenesis [20]. Furthermore, TAMs can educate and control invading leukocytes to promote angiogenesis, viability, motility, and invasion [21]. Additionally, the immunosuppressive activity of TAMs is performed indirectly by the secretion of chemokines that attract T cell subsets lacking cytotoxic function [12, 22]. It has been reported that the density of TAMs in many tumors correlates with increased angiogenesis, tumor invasion, and poor prognosis [23–25]. And numerous clinical studies support the protumorigenic role of TAMs in human cancers, showing significant correlation between elevated macrophage content and poor clinical outcome [23, 25, 26].

In the present study, high-monocyte count was positively associated with male, CEA level, and high tumor grade (Table 1), which suggested the potential interactions between them. As shown in the subgroup analysis, all of these covariates together with adjuvant therapy significantly affected the association of monocyte count with OS, but not DFS. One of the most important causes may be the small sample size of included patients and a few deaths. Actually, only CEA level was found to be strongly interacted with monocyte count for OS (*P* = 0.002). Since high CEA level is known to be an adverse prognosis in rectal cancer, thus the prognostic impact of monocyte count among patients with high CEA level, if existed, was likely to be covered by the effect of CEA level.

It is one of the limitations that this was a retrospective study with small sample size, which possibly caused skewed results; however, the included patients were restricted to those diagnosed with pathological T3N0M0, which lowered the uncertainty of staging by images. Moreover, the treatment heterogeneity, especially the influence of adjuvant chemotherapy, was another limitation. But this was included in the multivariate analysis and subgroup analysis, and resulted in small shift to the overall outcomes.

In conclusion, the absolute number of preoperative peripheral blood monocyte count is an independent predictive factor that can stratify the survival of rectal cancer patients staged with pathological T3N0M0 after TME resection. Postoperative adjuvant treatment might be considered for this sort of patients with high-monocyte count.

## Abbreviations

AR: Abdominoperineal resection
CRT: Chemoradiotherapy
CT: Computed tomography
DFS: Disease-free survival
LAR: Low anterior resection
MRI: Magnetic resonance imaging
OS: Overall survival
ROC: Receiver operating characteristic
TME: Total mesorectal excision
TAMs: Tumor-associated macrophages

## References

[1] Rodel C, Hofheinz R, Liersch T (2012). Rectal cancer: state of the art in 2012. Curr Opin Oncol.

[2] McCarthy K, Pearson K, Fulton R, Hewitt J (2012). Pre-operative chemoradiation for non-metastatic locally advanced rectal cancer. Cochrane Database Syst Rev.

[3] Sauer R, Becker H, Hohenberger W, Rodel C, Wittekind C, Fietkau R (2004). Preoperative versus postoperative chemoradiotherapy for rectal cancer. N Engl J Med.

[4] Hanahan D, Weinberg RA (2011). Hallmarks of cancer: the next generation. Cell.

[5] Wilcox RA, Ristow K, Habermann TM, Inwards DJ, Micallef IN, Johnston PB (2011). The absolute monocyte and lymphocyte prognostic score predicts survival and identifies high-risk patients in diffuse large-b-cell lymphoma. Leukemia.

[6] Sasaki A, Iwashita Y, Shibata K, Matsumoto T, Ohta M, Kitano S (2006). Prognostic value of preoperative peripheral blood monocyte count in patients with hepatocellular carcinoma. Surgery.

[7] Lee YY, Choi CH, Sung CO, Do IG, Huh S, Song T (2012). Prognostic value of pre-treatment circulating monocyte count in patients with cervical cancer: Comparison with scc-ag level. Gynecol Oncol.

[8] Schmidt H, Bastholt L, Geertsen P, Christensen IJ, Larsen S, Gehl J (2005). Elevated neutrophil and monocyte counts in peripheral blood are associated with poor survival in patients with metastatic melanoma: a prognostic model. Br J Cancer.

[9] Kumagai S, Marumo S, Shoji T, Sakuramoto M, Hirai T, Nishimura T (2014). Prognostic impact of preoperative monocyte counts in patients with resected lung adenocarcinoma. Lung Cancer (Amsterdam, Netherlands).

[10] Droin N, Hendra JB, Ducoroy P, Solary E (2009). Human defensins as cancer biomarkers and antitumour molecules. J Proteome.

[11] Parkin J, Cohen B (2001). An overview of the immune system. Lancet.

[12] Mantovani A, Allavena P, Sica A, Balkwill F (2008). Cancer-related inflammation. Nature.

[13] Grivennikov SI, Greten FR, Karin M (2010). Immunity, inflammation, and cancer. Cell.

[14] Bottazzi B, Polentarutti N, Acero R, Balsari A, Boraschi D, Ghezzi P (1983). Regulation of the macrophage content of neoplasms by chemoattractants. Science.

[15] Mantovani A, Schioppa T, Porta C, Allavena P, Sica A (2006). Role of tumor-associated macrophages in tumor progression and invasion. Cancer Metastasis Rev.

[16] Coussens LM, Werb Z (2002). Inflammation and cancer. Nature.

[17] Balkwill F, Mantovani A (2001). Inflammation and cancer: back to virchow?. Lancet.

[18] Coussens LM, Tinkle CL, Hanahan D, Werb Z (2000). Mmp-9 supplied by bone marrow-derived cells contributes to skin carcinogenesis. Cell.

[19] Locati M, Deuschle U, Massardi ML, Martinez FO, Sironi M, Sozzani S (2002). Analysis of the gene expression profile activated by the cc chemokine ligand 5/rantes and by lipopolysaccharide in human monocytes. J Immunol.

[20] Lewis CE, Leek R, Harris A, McGee JO (1995). Cytokine regulation of angiogenesis in breast cancer: the role of tumor-associated macrophages. J Leukoc Biol.

[21] Mantovani A, Germano G, Marchesi F, Locatelli M, Biswas SK (2011). Cancer-promoting tumor-associated macrophages: new vistas and open questions. Eur J Immunol.

[22] Sica A, Allavena P, Mantovani A (2008). Cancer related inflammation: the macrophage connection. Cancer Lett.

[23] Balkwill F (2004). Cancer and the chemokine network. Nat Rev Cancer.

[24] Bingle L, Brown NJ, Lewis CE (2002). The role of tumour-associated macrophages in tumour progression: implications for new anticancer therapies. J Pathol.

[25] Tsutsui S, Yasuda K, Suzuki K, Tahara K, Higashi H, Era S (2005). Macrophage infiltration and its prognostic implications in breast cancer: the relationship with vegf expression and microvessel density. Oncol Rep.

[26] Balkwill F, Charles KA, Mantovani A (2005). Smoldering and polarized inflammation in the initiation and promotion of malignant disease. Cancer Cell.

